# Light intensity affects RNA silencing of a transgene in *Nicotiana benthamiana *plants

**DOI:** 10.1186/1471-2229-10-220

**Published:** 2010-10-12

**Authors:** Christos Kotakis, Nicholas Vrettos, Dimitrios Kotsis, Mina Tsagris, Kiriakos Kotzabasis, Kriton Kalantidis

**Affiliations:** 1 Department of Biology, University of Crete, P.O. Box 2208, GR-71409 Heraklion, Crete, Greece; 2 Institute of Molecular Biology and Biotechnology, Foundation for Research and Technology - Hellas, P.O. Box 1527, GR-71110 Heraklion, Crete, Greece

## Abstract

**Background:**

Expression of exogenous sequences in plants is often suppressed through one of the earliest described RNA silencing pathways, sense post-transcriptional gene silencing (S-PTGS). This type of suppression has made significant contributions to our knowledge of the biology of RNA silencing pathways and has important consequences in plant transgenesis applications. Although significant progress has been made in recent years, factors affecting the stability of transgene expression are still not well understood. It has been shown before that the efficiency of RNA silencing in plants is influenced by various environmental factors.

**Results:**

Here we report that a major environmental factor, light intensity, significantly affects the induction and systemic spread of S-PTGS. Moreover, we show that photoadaptation to high or low light intensity conditions differentially affects mRNA levels of major components of the RNA silencing machinery.

**Conclusions:**

Light intensity is one of the previously unknown factors that affect transgene stability at the post-transcriptional level. Our findings demonstrate an example of how environmental conditions could affect RNA silencing.

## Background

RNA silencing pathways have been found to function in most eukaryotic organisms. Silencing induced by the expression of a transgenic sequence in the sense orientation is common in plants and is termed sense post-transcriptional gene silencing (S-PTGS) [1]. Some features of the transgenic mRNA that can trigger S-PTGS have been elucidated such as transgene copy number [2] and improper mRNA polyadenylation [3,4]. Nevertheless, it remains which factors affect whether an mRNA will induce silencing. It has been previously shown that environmental stimuli affect the RNA silencing mechanism in plants, from PTGS under temperature stress conditions [5] to small RNA populations showing seasonal oscillations [6]. Further studies revealed the involvement of temperature conditions in RNA silencing [7-11].

RNA silencing is initiated by the presence of double-stranded RNA (dsRNA) inside a cell [12]. In plants, dsRNA can also result from the activity of one of the RNA-DEPENDENT RNA POLYMERASEs (RDRs). RDR6 was one of the first S-PTGS indispensable enzymes to be identified, needed for the generation of dsRNA from a transgene [13,14]. It was later shown in grafting experiments that RDR6 is required for the perception and amplification of the silencing signal in scion responding tissues [15,16]. dsRNA is processed into 21-25nt small RNAs with the aid of RNAseIII-like enzymes called DICER or DICER-LIKE (DCL) [17]. *Arabidopsis thaliana *is reported to encode four *DCL *paralogues [18,19]. Small RNAs negatively regulate gene expression by guiding appropriate ARGONAUTE (AGO) structured effector complexes to complementary DNA or RNA [20]. AGO1 is a well studied member of the AGO family of proteins with an endonucleolytic activity in the cytoplasm [21]. It is involved in the S- and hairpin-PTGS (hp-PTGS) pathways but also in the biogenesis of miRNAs [reviewed by [22]].

Previously we reported the generation of green fluorescent protein (GFP) transgenic lines of *Nicotiana benthamiana *that, in a stochastic manner, induced silencing spontaneously at different frequencies and of different spreading intensities (short range versus systemic silencing) [23]. The frequency of induction of spontaneous silencing was strongly influenced by the genetic background (i.e. the transgenic line) of the plant. Nevertheless, plants of the same line grown in the greenhouse under controlled temperature showed significant variation in the frequency of spontaneous silencing when grown at different times. We considered this stochasticity as an indication of unknown, most likely, external factors affecting S-PTGS induction. The instability of the transgene expression in these plants could therefore serve as a sensitive system to identify such factors.

Plant ecophysiology is characterized by multiple regulatory mechanisms for acclimation under different environmental conditions. The light environment is crucial for the adaptation of plants. A central response to variations in both spectral quality and light intensity is the adjustment of the structure and function of the photosynthetic apparatus and therefore the photosynthetic capacity of the plant. The photoadaptation of plants to high and low light intensities is a well-documented phenomenon [reviewed by [24]].

Here we report that a major environmental factor, light intensity in physiological ranges, significantly affects the induction and systemic spread of S-PTGS in plants grown under stable temperature conditions. In addition, we show that photoadaptation of plants to high and low light conditions differentially affects mRNA levels of major components of the RNA silencing machinery such as DCL and RDR enzymes. Amongst them, *DCL4 *is found to display a light dependent induction profile even in the absence of a silencing trigger.

## Results

### Increased light intensity positively affects the frequency of spontaneous posttranscriptional gene silencing in transgenic plants

*N. benthamiana *GFP transgenic lines (line 5.1, line 5.3 and line 6.4) were grown under high and low light conditions. These lines carry two copies of a *GFP *transgene (see additional file 1: Figure S1 and [23]) and exhibit silencing initiation stochastically [23]. Silencing is initiated in single cells and either moves locally to 10-15 cells, a phenomenon previously termed spontaneous short-range silencing (SSRS) [23], or spreads systemically to the entire plant [25]. All three lines analyzed in this work exhibit SSRS while lines 5.1 and 6.4 display also spontaneous systemic silencing [23]. "High Light" intensity (HL) and "Low Light" intensity (LL) conditions used here, refer to 130 ± 20 μmol m^-2 ^s^-1 ^and 35 ± 15 μmol m^-2 ^s^-1 ^continuous white light, respectively. It should be emphasized that both light regimes do not impose stress (i.e. photoinhibition) on plants. Since temperature also influences silencing [7,8], we took care of keeping temperature stable throughout the course of our experiments in order to dissect the role of light in the silencing process. The temperature values did not differ more than 0.5°C under HL or LL conditions irrespectively of the distance of the plants from the light source (see additional file 2: Table S1). Leaves with fully suppressed GFP, as observed macroscopically by the lack of green fluorescence under UV light, were considered silenced. Leaves of the same plant which were fully fluorescing green under UV light were considered non-silenced. Each time SSRS or systemic silencing was observed on a plant, the phenotype and growth stage were scored and analysis pursued with the rest of the plants. As expected, plants that grew at the indicated LL conditions needed more time to reach the same leaf stage as HL plants. More specifically, HL-grown plants reached the 21-30 leaf stage in approximately 10 weeks, whereas it took 12 weeks for LL-grown plants to get to the same stage (see additional file 3: Figure S2). All the data presented here were categorized on a growth stage basis as estimated by number of leaves. Although the frequency and the extent of silencing occurrence differs according to the genetic background of each line, spontaneous systemic or short-range silencing emergence was always more frequent in plants grown under HL conditions, compared to the LL-grown ones in all the lines tested (see additional file 2: Table S2).

**Figure 1:**
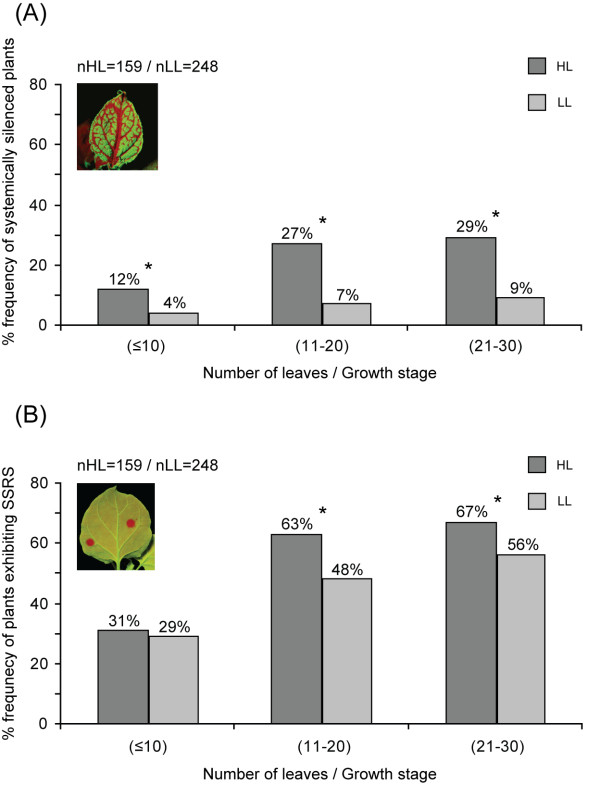
**Light intensity positively affects spontaneous silencing events in *Nicotiana benthamiana***. (A) Bars represent the frequency of spontaneous systemic silencing occurrence in plants (line 6.4) grown under HL and LL conditions. (B) Frequency of plants (line 6.4) that exhibited SSRS under HL and LL conditions. The leaf number is indicative of the growth stage at which silencing appeared. Leaf images at the top left represent the type of silencing monitored in each case. Asterisks (*) denote statistically significant difference. HL, high light intensity; LL, low light intensity; SSRS, spontaneous short-range silencing; nHL/LL, the total number of plants examined in each condition.

We chose to continue our study with one line (line 6.4) and examined silencing frequency values for plants of a specific genetic background that grew under HL or LL conditions at a stable temperature. The number of plants which exhibited systemic silencing was always significantly higher in all the examined growth stages, when plants grew under HL compared to LL conditions (Figure 1A). Based on this observation, we asked whether light accelerates specifically the process of silencing maintenance and spread and/or affects the onset of silencing. To address this, we monitored the percentage of plants that exhibited SSRS as this phenomenon indicates silencing initiation events failing to establish a systemic spread [23]. SSRS individual events appear as small GFP-silenced red spots under UV light. The HL-grown plants tended to exhibit SSRS at significantly higher frequency than LL plants, in the later than 10-leaf growth stages (Figure 1B). Overall these observations provide evidence that transgenic plants grown under HL intensity show significantly higher frequency of emergence for both silencing initiation and spread. Nevertheless, the difference in systemic silencing frequencies between HL and LL-grown plants (Figure 1A) is more pronounced than the corresponding difference for SSRS occurrence between the two light growth conditions (Figure 1B).

Next we wanted to address whether HL-grown plants tended to produce a higher density of silenced spots (more silenced spots per leaf area) when compared with LL-grown plants, as an indicator of independent silencing events. The average number of silenced spots per leaf area was calculated from five leaves collected from similar positions of individual HL and LL-grown plants exhibiting SSRS at the 21-30 leaf stage. The results showed no statistically significant difference in the number of silenced spots per leaf area between the two light growth regimes (see additional file 2: Table S3). These results suggest that light quantity affects the frequency of sensitization of the whole plant to silencing initiation rather than the actual independent silencing initiation events.

### Increased light intensity positively affects siRNA levels of S -and hp- PTGS

**Figure 2:**
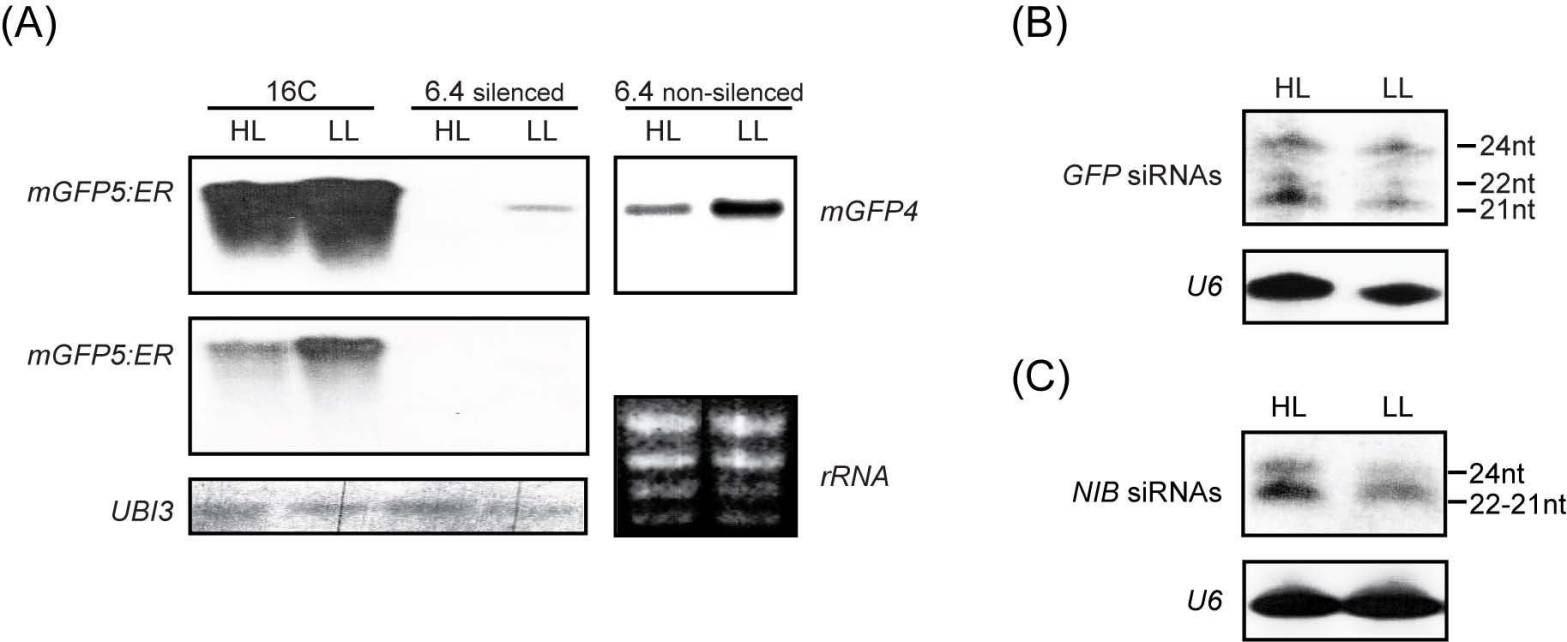
**HL-grown plants demonstrate higher silencing efficiency affecting both mRNA levels and siRNA production**. (A) Northern blot detection for *mGFP5:ER *and *mGFP4 *in 16C, 6.4 fully silenced and 6.4 non-silenced leaf tissue, from plants grown under HL and LL conditions. 6 h and 2.5 h of autoradiographic exposure time were applied in the higher and middle panels, respectively. *UBI3 *transcripts and 18S/chloroplast *rRNA *ethidium bromide staining served as loading controls. (B) Detection of *GFP*-siRNAs produced under HL and LL in fully silenced leaf tissue. (C) Detection of *Nib*-siRNAs derived from an hp*Nib*RNA in plants grown under HL and LL. *U6 *probing was used as loading control (lower panels). GFP, green fluorescent protein; HL, high light intensity; LL, low light intensity; UBI3, ubiquitin; Nib, *Plum pox virus *polymerase; hp, hairpin.

We tested whether plants grown under HL and LL conditions differ at the transgene RNA level. To address this, the *GFP *mRNA levels were analyzed by Northern hybridizations. We collected and pooled leaf material from at least five 21-30 leaf stage plants from each light regime (HL and LL). Tissue was sampled from fully silenced and non-silenced branches of the 6.4 line along with leaves from the *N. benthamiana *16C line. The latter represents an additional negative control for our study since 16C line plants stably express GFP and hardly undergo any type of spontaneous silencing [26]. Northern blot analysis revealed that LL-grown plants coming from either the 16C GFP expressing line or non-silenced 6.4 line tissue displayed higher amounts of *GFP *transcripts in comparison to the HL-grown corresponding plants (Figure 2A, lanes 1,2,5,6). Interestingly the LL-grown silenced leaf samples maintained a small quantity of *GFP *transcripts (Figure 2A, lanes 3,4) although GFP fluorescence could not be detected macroscopically under UV light (not shown). Taken together these results indicate that transgene mRNA levels are negatively affected by HL intensity in both silenced and GFP expressing tissue.

Next, we analyzed the effect of light on the levels of small interfering RNAs (siRNAs) which are the hallmark of RNA silencing [27]. In plants three distinct siRNA size classes exist of 21nt, 22nt and 24nt, which are generated by the activity of DCL4, DCL2 and DCL3 respectively [28,29]. Northern blot analysis revealed the siRNA steady state levels in HL and LL-grown fully silenced leaves from line 6.4. We observed a moderate increase in the amount of siRNAs from all three classes in fully silenced leaf tissue from HL conditions in comparison to LL (Figure 2B). Next, we pursued with detecting the siRNAs produced by a transgenic line that underwent hairpin induced PTGS. For this purpose *N. benthamiana *line 20-1A1 engineered to express a hairpin for a *Nib *gene fragment taken from *Plum pox virus *(PPV), was treated with the same light conditions as for the sense-silencing inducing 6.4 line. In this case dsRNA is directly transcribed inside the plant cell without a need for the RDR6 processing step [30]. The total amount of siRNAs detected was higher when the hairpin-plants were grown under HL conditions with apparent differences in all distinguishable siRNA classes (Figure 2C). These results indicate that light intensity also affects RNA silencing efficiency at steps downstream of dsRNA formation.

### Increased light intensity significantly affects mRNA levels of key enzymes of the RNA silencing pathways

With the aim of identifying potential homologues of defined *AtDCL *genes in *N. benthamiana*, we searched the International Solanaceae Genome Project database http://www.sgn.cornell.edu[31] for relevant ESTs. Several candidate sequences emerged from the tomato, potato and tobacco EST collections. These gene fragments were aligned with the *AtDCL *sequences and the most conserved regions were selected for PCR primer design. The corresponding *DCL1*, *DCL2*, *DCL3 *and *DCL4 *gene fragments were successfully amplified from *N. benthamiana *cDNA and further sequenced and certified through BLAST analysis (see additional file 2: Table S4). The cloned *NbDCL1*, *NbDCL2*, and *NbDCL4 *fragments encoded a part of the second RNAseIII domain, whereas the *NbDCL3 *fragment corresponded to a part of the second dsRNA binding domain. Each *NbDCL *sequence shared higher homology with the corresponding *A. thaliana *orthologue than with any of the related paralogues (see additional file 2: Table S5). Our *NbDCL *cDNA fragments for *DCL1 *and *DCL2 *are in full agreement with the corresponding *NbDCL *fragments published elsewhere during the revision of this manuscript [32]. In the same work the provided *NbDCL3 *and *NbDCL4 *sequences corresponded to different regions of the coding sequence than the fragments used in this study. In order to be able to compare our sequences to the published ones, we performed RT-PCR reactions where our primers were combined with those from Kuang et al. [32]. The results strongly indicated that both our sequences and the published ones are fragments of the same *DCL3 *or *DCL4 *transcripts, respectively (not shown).

**Figure 3:**
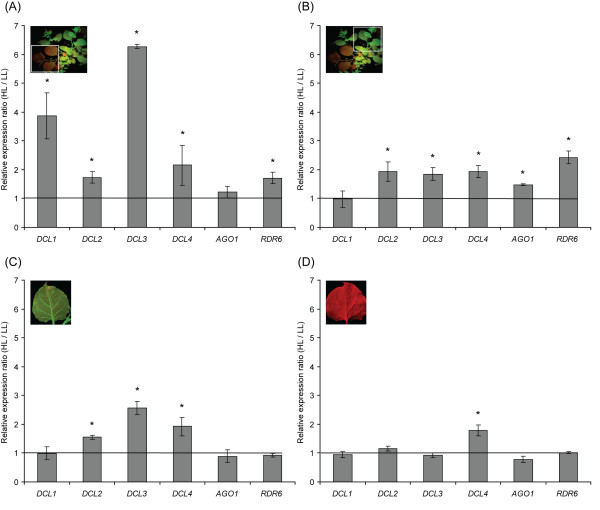
**High light conditions favour the upregulation of several silencing related genes**. Real-time quantitative PCR analysis revealed the relative expression ratio for *Nicotiana benthamiana DCL1*, *DCL2*, *DCL3*, *DCL4*, *AGO1*, and *RDR6 *assayed at HL over LL conditions for different silencing states or genetic backgrounds. (A) Fully silenced leaf tissue from line 6.4. (B) Non-silenced leaf tissue from line 6.4 where silencing is established in neighbouring branches. (C) Leaf tissue from line 16C. (D) Wild type leaf tissue. The horizontal line designates the zero-change level in both light regimes (ratio one). Images at the top left depict the type of leaf tissue used in each experiment. Asterisks (*) denote statistically significant difference. DCL, dicer-like; AGO1, argonaute 1; RDR6, RNA-dependent RNA polymerase 6; HL, high light intensity; LL, low light intensity.

We investigated the mRNA levels of major silencing related genes. This analysis encompassed the *N. benthamiana *orthologues for the *A. thaliana DCL1-4 *plus the *NbRDR6 *[reviewed by [33]] and *NbAGO1 *[34] genes. mRNA levels were assessed with quantitative real-time PCR (qPCR). qPCR analysis was performed in cDNA preparations generated from pooled leaf tissue of at least five plants in each case. In order to avoid effects of leaf growth stage on gene expression, leaves of similar stage were mixed and used for the RNA extractions. The results of qPCR were analyzed and presented in a pairwise relative ratio for HL over LL conditions in wild type (wt), 16C and 6.4 leaf tissue (Figure 3, see additional file 2: Table S6).

Plants of line 6.4 at the process of spontaneous systemic silencing offered the opportunity to monitor the mRNA levels of major genes of the silencing pathways in silenced and non-silenced tissue from the very same plant. Analysis of the 6.4 line silenced leaf material grown under HL conditions by qPCR revealed that the expression levels of *DCL1 *and *DCL3 *in these plants were approximately 3.9 and 6.3 times higher, respectively than for the LL-grown plants (Figure 3A). The other genes tested, displayed a moderate increase under HL (*DCL2*, *DCL4 *and *RDR6*) or no significant change (*AGO1*) (Figure 3A).

In non-silenced tissue from the same 6.4 plants with ongoing silencing in other parts of the plant (see image on top left of Figure 3A,B), all the genes under study were found to be upregulated by a factor of approximately 2 under HL with *RDR6 *and *DCL3 *exhibiting a 2.4 and 1.8 increase respectively (Figure 3B), except for *DCL1 *which remained unaltered (Figure 3B). It should be noted that although this material was isolated from phenotypically non-silenced tissue it is possible that silencing signals that were present in these plants might be affecting silencing related genes and therefore this tissue cannot be considered as non-silenced material in a strict sense. For this reason, tissue from a stably GFP-expressing line (16C) that does not undergo silencing spontaneously was also analyzed. Analysis from 16C line tissue (HL over LL), revealed approximately 2.6, 1.9 and 1.6 fold increase of *DCL3*, *DCL4 *and *DCL2 *respectively (Figure 3C). *AGO1, DCL1 *and *RDR6 *were not significantly affected by light intensity (Figure 3C). Interestingly *DCL3 *was found to be upregulated in all cases where transgenic lines were assayed irrespectively of the silencing status (Figure 3A-C). Finally, analysis from tissues of wt plants showed that the mRNA levels of *DCL1, DCL2 DCL3, AGO1 and RDR6 *did not exhibit a light-responsive profile (Figure 3D). By contrast, *DCL4 *which is reported to be the first *DCL *gene to be activated upon dsRNA presence [28], exhibited an approximately 2-fold increase under HL in all the genetic backgrounds and silencing states tested (Figure 3A-D).

### Evidence for the involvement of blue light photoadaptation in elevated frequency of S-PTGS

The surrounding light perceived by photosynthetic organisms can highly vary in intensity and quality. Plants have to cope with this dynamic light environment by strategies that involve changes in the composition and function of the photosynthetic machinery [24]. The effect of light quality on the RNA silencing mechanism described above may be related to a differential acclimation of the photosynthetic apparatus to a dynamic light environment [35,36], or could be a photosynthesis independent mechanism. In order to distinguish between these two possibilities the response of silencing initiation and spread was also studied under different light qualities. It is known that blue and red light respectively confer HL- and LL-adaptation of the photosynthetic apparatus in higher plants [37,38]. If silencing frequencies differed in blue and red light conditions, this would indicate that the acclimation response of the photosynthetic apparatus and silencing are related processes.

In this context, line 6.4 was grown under blue and red spectral light (see Methods section, simply referred onwards to blue and red light respectively) and exposed to the same amount of light intensity (18 ± 5 μmol m^-2 ^s^-1^) while the temperature was kept stable. Under such limiting light intensity conditions plants developed at a slow pace reaching the stage of approximately 5 leaves in 10 weeks time, while the corresponding white light grown plants (LL conditions) were already approaching the 20-leaf stage. From this stage onwards plants started to develop chlorotic symptoms and signs of physiological decline that did not allow further analysis. Plants grown under blue light in comparison to red light grown plants, showed a higher frequency of systemic silencing although this difference was not found to be statistically significant (see additional file 2: Table S7). The 6.4 line response to a blue light regime with elevated silencing frequencies is in accordance to the effect of HL conditions on silencing initiation and spread (see above). The frequency of plants exhibiting SSRS was 11% for blue light grown plants whereas none of the red light grown plants was found to have silenced spots at this early growth stage (see additional file 2: Table S8). Taken together these results suggest that the photoadaptive status of the photosynthetic apparatus and RNA silencing may be correlated.

## Discussion

### Light affects RNA silencing

Transgene silencing is a phenomenon still not well understood. Gene sequences even of endogenous origin, when expressed as transgenes, often trigger silencing in a spontaneous manner. Some factors affecting this event have been identified mostly originating from the transgenesis process. Additionally to intrinsic factors, a substantial environmental parameter, temperature, was found to affect siRNA production and consequently RNA silencing efficiency [7,8]. Here, we present evidence that another major environmental factor, light intensity at physiological ranges, affects RNA silencing pathways.

There are various reports in the literature implicating that light affects RNA silencing induction and spread. Vaucheret *et al*. [39] reported a strong seasonal effect on the initiation of silencing of a *NITRATE REDUCTASE *transgene in tobacco plants. In addition, high light conditions have been shown to favor the maintenance of virus induced gene silencing for *PHYTOENE DESATURASE *[40]. However, in these experiments the effect of temperature was not isolated from the effects of light on silencing initiation and maintenance. Experiments presented here were carefully set up with the aim of excluding the impact of temperature on the analysis and study solely the influence of different light regimes.

The use of GFP transgenic lines that show stochastic frequencies of silencing initiation and spread enabled us to investigate effects that light may have on silencing events. Three different lines were used for the study of spontaneous silencing occurrence with comparable results indicating that the effect of light on silencing is not restricted to a specific insertional event. We found that HL intensity increased the frequency of plants undergoing both short range and systemic silencing. LL plants exhibited a moderately slower growth rate than HL plants; nevertheless they still exhibited lower silencing frequencies even when they reached the same growth stage as HL plants. Hence, the lower silencing frequencies observed under LL could not be attributed to the slower growth rate.

The number of plants that underwent SSRS in this study increased under HL conditions although the number of individual non-spreading silencing events per leaf area did not. It is possible that light intensity affects the frequency of sensitization of the whole plant to silencing initiation. Once a plant has become sensitized (whether under HL or LL), silencing initiation events occur in a comparable frequency in both light regimes. From the present study it is apparent that the difference in systemic silencing frequencies between HL- and LL-grown plants is more pronounced than the corresponding difference for SSRS occurrence between the two light growth conditions. Differences in systemic silencing under HL conditions could spring from more than one source such as increased silencing initiation and signal generation, increased sensitivity of signal perception, or increased conductivity for the signal under HL conditions. Although we did not distinguish between these possibilities our qPCR analysis hints that genes related to signal perception and dsRNA processing are indeed upregulated (see below).

It is established that blue and red light respectively confer HL- and LL-adaptation of the photosynthetic apparatus in higher plants [37,38]. Moderate differences in the frequency of S-PTGS under blue and red light conditions were detected and this may indicate that the photoadaptive status of the photosynthetic machinery and silencing are correlated. A percentage of plants grown under red light developed systemic silencing while no red light treated plant exhibited SSRS. The factors controlling the SSRS phenomenon remain unknown [23], but we favor the possibility that light intensity is one of them. It seems that under light of such quality (red), spontaneous silencing initiation events occur rarely and/or when they do occur, they are rapidly extended to systemic silencing. Furthermore, acclimation to high light conditions reinforces the plant's defense in upcoming stress signals [41]. Silencing is a vital defense response to invading exogenous nucleic acids [42], which we could call stress-imposing factors. It is plausible that changes in the photosynthetic metabolism due to stress conditions [43,44] may provide signals that regulate the differential frequency of silencing occurrence. Nevertheless, further work is needed in order to reveal the interactions between RNA silencing and photosynthesis.

### The molecular signature of light on RNA-silencing

When S-PTGS took place under HL conditions, 6.4 line plants displayed moderately higher amounts of siRNAs when compared to LL-grown plants. Furthernore, the accumulation of siRNAs produced by an unrelated hp-generating transgene was more pronounced in HL-grown plants. We also observed increased mRNA levels for several silencing related key enzymes. Therefore at least one light affected step appears to exist downstream of dsRNA generation.

It is not clear how light intensity could similarly affect the *GFP *mRNA levels both in 16C and 6.4 line non-silenced samples. One interpretation could be that the activity of the 35S *Cauliflower mosaic virus *(CaMV) transgene promoter is affected by light, since CaMV infections are more pronounced when light intensity is reduced [45,46]. Alternatively, it is possible that a small degree of GFP silencing is taking place in 6.4 and 16C non-silenced plants which does not reach a threshold level and therefore fails to be amplified [23]. This background GFP silencing is apparently stronger under HL conditions resulting in lower levels of *GFP *mRNAs.

In order to dissect the effect of light intensity on RNA silencing we monitored the relative mRNA levels of *DCL1-4*, *RDR6 *and *AGO1 *in different lines or silencing states treated with HL and LL. According to our real time PCR analysis the silencing related genes studied here fall into three different categories: *DCL4*, which displayed a light responsive profile in all the cases studied including wt plants. *DCL1-3 *and *RDR6*, which were highly or moderately induced under HL provided that silencing had initiated in local or distant parts of the same plant. Their expression under HL in wt plants though, remained unchanged. Finally, *AGO1*, whose expression was not changed more than 1.5 fold under HL in all the cases studied. Therefore, silencing related genes respond to HL and ongoing silencing in a distinct and repetitive pattern, rather than display a generalized light-driven induction profile.

In systemically silenced tissue studied here, *DCL1 *mRNA levels were found to be strongly affected upon HL growth. Apart from its well characterized role in the biogenesis of miRNAs [19] it was shown in *A. thaliana *that DCL1 facilitates the production of DCL3 and DCL4 dependent siRNAs originating from inverted repeat transgenes [47]. It is likely that DCL1 holds a similar role in S-PTGS. On the other hand, qPCR analysis in HL-grown non-silenced leaf tissue where silencing is established in neighboring branches, disclosed an induction profile for *RDR6 *and *DCL3*, when compared with LL samples. Grafting experiments have shown that both genes are essential for the establishment of long distance silencing spread in *A. thaliana*, facilitating or enabling the perception of the systemic signal [16]. Finally it should be noted that in the tissues of transgenic origin analyzed, *DCL3 *was found to be significantly upregulated. DCL3 has been primarily implicated in epigenetic related phenomena and RNA-dependent DNA Methylation [reviewed by [48]]. Given the fact that in 16C and 6.4 line non-silenced samples we observed a decrease at the *GFP *mRNA levels under HL conditions; we speculate that the increase in the *DCL3 *mRNA is related to an epigenetic decrease of transgene expression in all the lines tested.

Interestingly *DCL4 *was positively affected with similar fold levels by HL in all types of tissue studied. This fact coincides with recent *in silico *data where the *DCL4 *promoter was found enriched with 3-6 fold more light responsive elements than any other *DCL *promoter in *Arabidopsis*, rice, grape [49] and tobacco (our unpublished data). Given the primary role of DCL4 in antiviral response [28] it is tempting to speculate that light induced *DCL4 *upregulation could represent a first aid defense system against occasional virus threats or be connected with rising populations of small RNAs of endogenous origin. It is well established that RNA silencing contributes significantly to the antiviral defense of plants [reviewed by [50]]. Our findings are in agreement with older virological observations where plants exposed to reduced light intensity became more susceptible to virus infections [45]. Furthermore, it had been shown that light intensity and quality influence the number of local lesions caused by plant viruses [51,52].

This work uncovers the important role of light intensity on the frequency of silencing events of transgenes. Given the generic induction profile of *DCL4 *and the central role it holds in the S-PTGS pathway, it is tempting to speculate that this gene is partly responsible for the increased frequency of silencing observed. While silencing spreads inside a plant grown under HL, all four *DCL *genes are upregulated enabling higher silencing rates, whereas *DCL3 *and *RDR6 *induction in not yet silenced tissues facilitate the rapid perception and amplification of the systemic silencing signal.

## Conclusions

In summary we report that plants tend to exhibit higher silencing frequencies under HL, and when this is evident, they also demonstrate higher silencing potency. Light intensity is one of the previously unknown factors that affect transgene stability at the post-transcriptional level. Our findings demonstrate an example of how environmental conditions could affect RNA silencing. Since transgenic plants could be grown in areas with quite different light regimes across the world, the effect of such a major environmental factor on RNA silencing may also be of practical interest. LL conditions should be applied in order to achieve stable transgene expression and protection from viral infections. Conversely, knock-down strategies based on RNA silencing would require HL conditions.

## Methods

### Plant Material

Lines 5.1, 5.3 and 6.4 were described previously [23]. Line 16C was kindly provided by David Baulcombe (University of Cambridge, UK). *N. benthamiana *20-1A1 transgenic line engineered to express a hairpin for *Nib *gene fragment from PPV was also used. Seeds germinated on MS medium [53] and plantlets were transplanted into soil at the cotyledon stage. Plantlets were kept under LL conditions, for 10 days, before each light regime was applied. Plants were grown in a chamber of 70% relative humidity and 22 ± 0.5°C temperature. Illumination was provided as continuous white light under a panel of cool-white fluorescent tubes (TL-D, 50 W/84o HF, Electronic NG, Phillips, Holland) at a photosynthetically active radiation (PAR: 400-700 nm). Experiments of spectral quality were performed in the same light-tight room, under red or blue light obtained through a filter sheet (Plexiglass GS Rot 501 or GS Blau 610, 3 mm thick; Rohm GmbH, http://www.rohm.com). Irradiance and temperature were preliminary measured with a QRT1 quantum sensor (Hansatech Instruments, http://www.hansatech-instruments.com).

### Phenotypic analysis and statistics

Transgenic *N. benthamiana *plants were checked for GFP fluorescence using a handheld 1000 W long-wavelength UV lamp (B100AP; Ultraviolet Products, http://www.uvp.com). Two-sample independent t-test (confidence intervals: 5%) was applied in order to check whether light treatment and spontaneous silencing phenotype occurrence are statistically significant (Figure 1). Statistical significance between light treatment and number of spots per leaf area (see additional file 2: Table S3), was checked also with the method above. SPSS 16.0 statistical package (SPSS Inc, http://www.spss.com) was used for statistical analysis.

### *Nib*-hairpin construction and plant transformation

A 1577 bp *Sac*I-*Bam*HI cDNA fragment encoding the *Nib *gene from a greek PPV strain (nucleotides 8022-8580 of the PPV genome, Acc. No PPV-DX16415.1) was cloned in pT3T7 (Boehringer Mannheim) in (+) orientation. A 1,444 bp λ-phage DNA fragment (corresponding to nucleotides 31,301 to 32,745) was introduced in the above plasmid at the *Acc*I restriction site, serving as spacer sequence for the hairpin. Both inserts (*Nib*+ and λ-spacer) were subcloned into another pT3T7 plasmid containing a 503 bp *Hind*III fragment of the 3" coding sequence of the *Nib *gene (nucleotides 8529-2022) in (-) orientation, in *Sac*I - *Sph*I restriction sites. The resulting plasmid was partially digested with *Hind*III in order for a 2546 bp fragment to be excised which contained 503 bp of the *Nib *sequence in opposite orientations separated by the λ-spacer (hairpin cassette). The *Nib*-hairpin cassette was subcloned into the binary pART7/27 vector system under the control of CaMV 35S promoter [54]. The final plasmid pART27PPVPH was introduced in *Agrobacterium tumefaciens *strain LBA4404 via triparental mating [55]. *N. benthamiana *transformation was performed exactly as described previously for *N. tabacum *[7].

### RNA preparation and Northern blot

RNA isolation was performed according to Papaefthimiou *et al*. [56]. Plasmids harbouring *mGFP4*, *Nib*, the mouse *U6 *snRNA gene and *NbUBI3 *were used for the generation of probe-templates. Northern blot analyses were conducted according to previous lab publications [7,56] with minor modifications. Membranes were incubated in church buffer (Sodium Phosphate Buffer 0,25 M, pH 7.2, 1 mM EDTA, 1% BSA, 7% SDS) at 65°C and 42°C for the detection of mRNAs and siRNAs, respectively.

### Quantitative Real-Time PCR

1.6 μg of DNAseI (Roche, http://www.roche-applied-science.com) treated RNA was reverse transcribed (SUPER RT, HT Biotechnology Ltd, Cambridge, UK) with an oligo-dT primer or a gene specific primer for the case of *NbDCL1*. The cDNA mix was diluted 10 times (5 times for *NbDCL1*) and 5 μl were used at the subsequent qPCR. Reactions were performed in an Opticon cycler (MJ Research, Waltham MA, USA) using the Sybr-Green method according to the following protocol: 1 cycle at 94°C for 5 m; 36 cycles of 94°C for 30s, 58-60°C for 30s and 72°C for 30s. The PCR primers (see additional file 2: Table S9) were designed with OLIGO 6 (Molecular Biology Insights Inc, http://www.oligo.net). Results were normalized against *UBIQUITIN *(*UBI-3*) and *ELONGATION FACTOR-1 ALPHA *(*EF-1*) genes [57]. Samples were processed in triplicates and every PCR run was repeated at least 2 times. The obtained data were analyzed according to Pfaffl *et al*. [58] and statistical significance was tested with one way ANOVA (P < 0.05). SigmaStat 3.5 statistical software package (Systat Software Inc, http://www.sigmaplot.com) was used for statistical analysis.

Sequence data from this article were submitted to the EMBL Nucleotide Sequence Database under the accession numbers: [FM986780 (NbDCL1), FM986781 (NbDCL2), FM986782 (NbDCL3), FM986783 (NbDCL4)].

## Authors' contributions

CK and NV carried out the experiments participated in designing the study and wrote the manuscript. DK and MT contributed to the experimental work and the manuscript preparation, respectively. KKo and KKa conceived of and coordinated the study, designed the experiments and participated in manuscript preparation. All authors read and approved the final manuscript.

## Supplementary Material

Additional file 1 **Supplementary Figure 1**. TIFF Figure S1 - Southern hybridization of line 6.4. Sample was digested with *Sac*I and separated on a 0.8% gel before being transferred to the membrane. A DNA *GFP *full sequence probe was used. Click here for file

Additional file 2 **Supplementary Tables**. PDF Table S1 - Temperature values (average ± standard deviation) in °C, taken from the leaf surface of plants grown under high and low light conditions. Fifty temperature measurements were performed in each case. Table S2 - Number of plants exhibiting spontaneous systemic silencing or spontaneous short-range silencing (SSRS) under high and low light conditions over the total number of plants examined (5.1 and 5.3 line). Table S3 - Number of silencing spots (SSRS events) over cm^2 ^of leaf area (average ± standard deviation) appeared on plants grown under high and low light conditions (6.4 line). Table S4 - Sequence homology values (%) between *N. benthamiana DCL *gene fragments and the corresponding *A. thaliana DCL *orthologue. Table S5 - Amino-acid identity and similarity values (%) between *N. benthamiana *DCL fragments and *A. thaliana *DCL1-4. Table S6 - Relative expression ratio values (average ± standard deviation) for high over low light grown plants as determined by real-time qPCR analysis in different types of leaf tissue. Table S7 - Number of plants exhibiting spontaneous systemic silencing under blue and red light conditions over the total number of plants examined (6.4 line, ≤5 leaf stage). Table S8 - Number of plants exhibiting spontaneous short-range silencing (SSRS) under blue and red light conditions over the total number of plants examined (6.4 line, ≤5 leaf stage). Table S9 - List of primer sequences used in quantitative real-time PCR assays. Click here for file

Additional file 3 **Supplementary Figure 2**. TIFF Figure S2 - LL-grown plants need approximately 2 weeks more time than HL plants, in order to reach the 20-30 leaf stage. Growth curve of plants grown under HL and LL conditions. HL, high light intensity; LL, low light intensity; nHL/LL, the total number of plants examined in each condition. Click here for file
