# Supplementary figures and images for: Light intensity affects RNA silencing of a transgene in Nicotiana benthamiana plants
Source: BMC Plant Biol. 2010 Oct 12;10:220. doi: 10.1186/1471-2229-10-220 (PMC3017829; doi:10.1186/1471-2229-10-220)

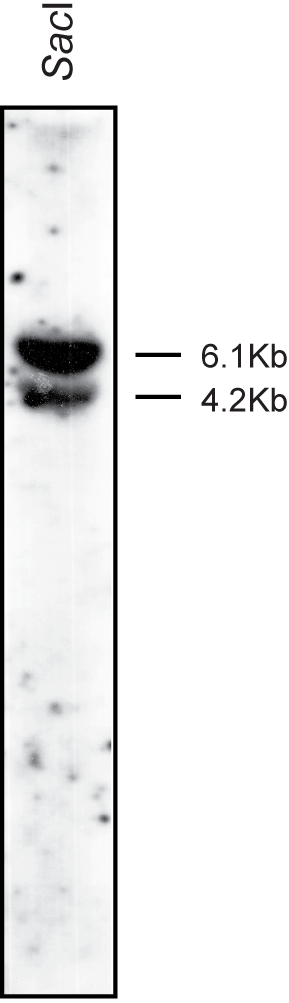

Supplement: Additional file 1 — Supplementary Figure 1. TIFF Figure S1 - Southern hybridization of line 6.4. Sample was digested with SacI and separated on a 0.8% gel before being transferred to the membrane. A DNA GFP full sequence probe was used. [file 1471-2229-10-220-S1.TIFF]

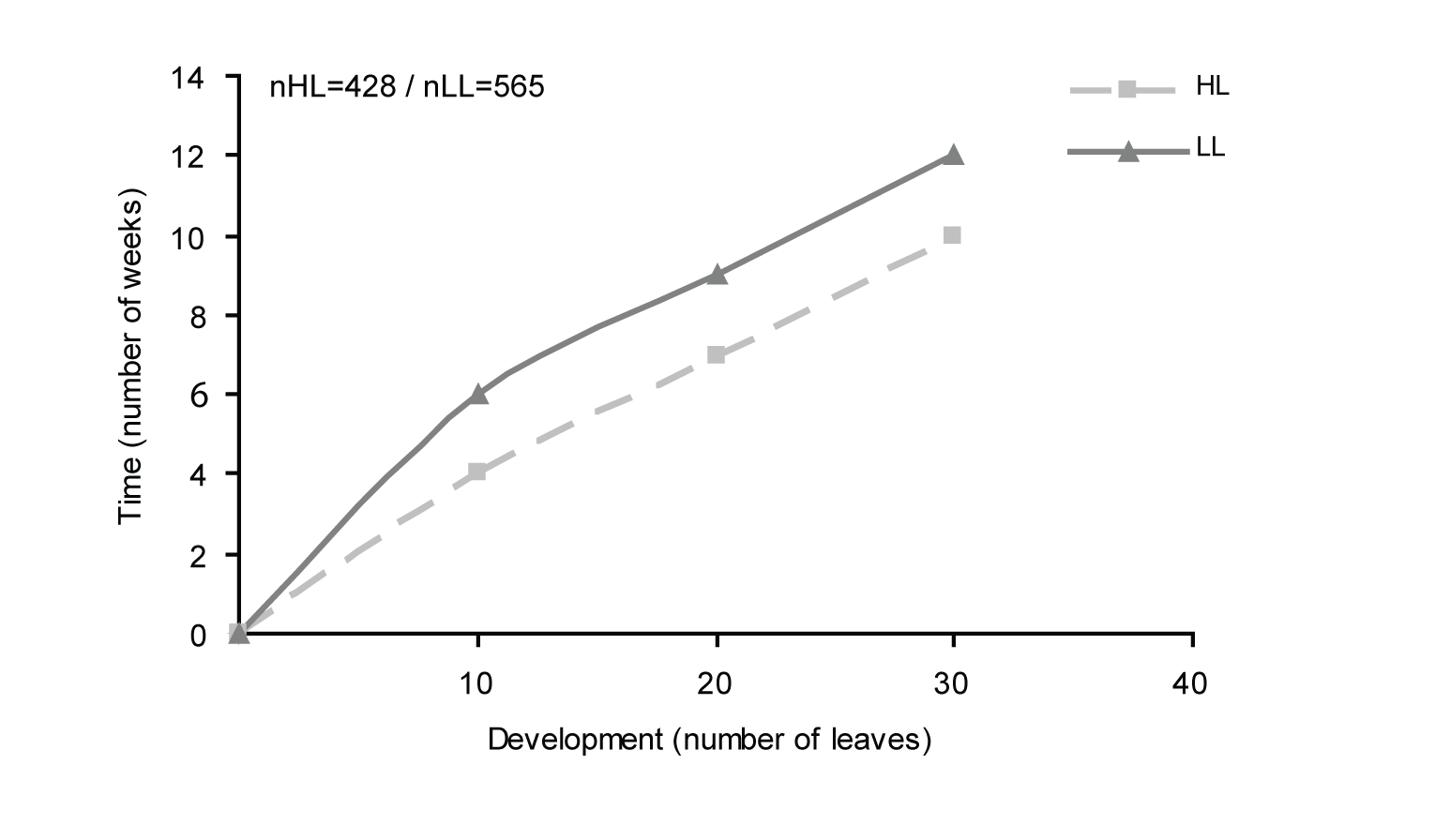

Supplement: Additional file 3 — Supplementary Figure 2. TIFF Figure S2 - LL-grown plants need approximately 2 weeks more time than HL plants, in order to reach the 20-30 leaf stage. Growth curve of plants grown under HL and LL conditions. HL, high light intensity; LL, low light intensity; nHL/LL, the total number of plants examined in each condition. [file 1471-2229-10-220-S3.TIFF]
