# Supplementary material for: Light intensity affects RNA silencing of a transgene in Nicotiana benthamiana plants
Source: BMC Plant Biol. 2010 Oct 12;10:220. doi: 10.1186/1471-2229-10-220 (PMC3017829; doi:10.1186/1471-2229-10-220)
Supplement: Additional file 2 — Supplementary Tables. PDF Table S1 - Temperature values (average ± standard deviation) in °C, taken from the leaf surface of plants grown under high and low light conditions. Fifty temperature measurements were performed in each case. Table S2 - Number of plants exhibiting spontaneous systemic silencing or spontaneous short-range silencing (SSRS) under high and low light conditions over the total number of plants examined (5.1 and 5.3 line). Table S3 - Number of silencing spots (SSRS events) over cm2 of leaf area (average ± standard deviation) appeared on plants grown under high and low light conditions (6.4 line). Table S4 - Sequence homology values (%) between N. benthamiana DCL gene fragments and the corresponding A. thaliana DCL orthologue. Table S5 - Amino-acid identity and similarity values (%) between N. benthamiana DCL fragments and A. thaliana DCL1-4. Table S6 - Relative expression ratio values (average ± standard deviation) for high over low light grown plants as determined by real-time qPCR analysis in different types of leaf tissue. Table S7 - Number of plants exhibiting spontaneous systemic silencing under blue and red light conditions over the total number of plants examined (6.4 line, ≤5 leaf stage). Table S8 - Number of plants exhibiting spontaneous short-range silencing (SSRS) under blue and red light conditions over the total number of plants examined (6.4 line, ≤5 leaf stage). Table S9 - List of primer sequences used in quantitative real-time PCR assays. [file 1471-2229-10-220-S2.PDF]

Supplementary Tables

Table S1. Temperature values (average ± standard deviation) in ^{o}C, taken from the leaf surface of plants grown under high and low light conditions. Fifty temperature measurements were performed in each case.

Light conditions	Leaf temperature (^{o}C)
High Light	21.542 ± 1.597
Low Light	21.747 ± 1.549

Table S2. Number of plants exhibiting spontaneous systemic silencing or spontaneous short-range silencing (SSRS) under high and low light conditions over the total number of plants examined (5.1 and 5.3 line).

Light conditions	Number of plants exhibiting systemic silencing / total number of plants examined (%) 5.1 line		
	(≤10)#	(11-20)#	(21-30)#
High Light	20/179 (11%)	41/179 (23%)	42/179 (24%)
Low Light	7/191 (4%)	18/191 (9%)	20/191 (11%)
**Light conditions**	**Number of plants exhibiting SSRS / total number of plants examined (%) 5.1 line**		
	(≤10)#	(11-20)#	(21-30)#
High Light	48/179 (27%)	127/179 (71%)	129/179 (72%)
Low Light	38/191 (20%)	83/191 (44%)	99/191 (52%)
**Light conditions**	**Number of plants exhibiting SSRS / total number of plants examined (%) 5.3 line**		
	(≤10)#	(11-20)#	(21-30)#
High Light	56/90 (62%)	78/90 (87%)	84/90 (93%)
Low Light	25/126 (20%)	92/126 (73%)	101/126 (80%)

Number of leaves indicative of the growth stage.

Table S3. Number of silencing spots (SSRS events) over cm^2 of leaf area (average ± standard deviation) appeared on plants grown under high and low light conditions (6.4 line).

**Light conditions**	**Number of spots / cm^2**
High Light	0.087 ± 0.014
Low Light	0.107 ± 0.009

Table S4. Sequence homology values (%) between *N. benthamiana DCL* gene fragments and the corresponding *A. thaliana DCL* orthologue.

***N. benthamiana DCL* gene fragment**	**Nucleotide identity**	**Amino-acid identity**	**Amino-acid similarity**
DCL1	76.7 %	89.9 %	93.7%
DCL2	64.6 %	63.8 %	75.9%
DCL3	58.5 %	51.5 %	72.2%
DCL4	63.6 %	51.7%	65.5%

Table S5. Amino-acid identity and similarity values (%) between *N. benthamiana* DCL fragments and *A. thaliana* DCL1-4.

***N. benthamiana* DCL gene fragment**	**Amino-acid identity/similarity with the *A. thaliana* DCL**			
	AtDCL1	AtDCL2	AtDCL3	AtDCL4
DCL1	**89.9%/93.7%**	55.1%/71.0%	61.2%/77.6%	45.6%/61.8%
DCL2	36.5%/50.2%	**63.8%/75.9%**	40.2%/54.2%	38.8%/55.5%
DCL3	30.5%/45.3%	30.4%/50.0%	**51.5%/72.2%**	29.0%/42.0%
DCL4	31.6%/50.4%	25.7%/54.5%	29.3%/46.4%	**51.7%/65.5%**

The cloned *NbDCL1*, *NbDCL2*, and *NbDCL4* gene fragments encode a part of the second RNAseIII domain, whereas the *NbDCL3* fragment corresponds to a part of the second double-stranded RNA binding domain.

Table S6. Relative expression ratio values (average ± standard deviation) for high over low light grown plants as determined by real-time qPCR analysis in different types of leaf tissue.

**Type of leaf tissue**	**Gene of Interest**					
	DCL1	*DCL2*	*DCL3*	*DCL4*	*AGO1*	*RDR6*
GFP silenced (6.4 line)	3.869 ± 0.792	1.734 ± 0.201	6.273 ± 0.069	2.152 ± 0.723	1.213 ± 0.182	1.704 ± 0.200
GFP non-silenced (6.4 line)	0.982 ± 0.278	1.937 ± 0.332	1.813 ± 0.216	1.937 ± 0.206	1.487 ± 0.023	2.426 ± 0.222
GFP stably expressing (16C line)	0.996 ± 0.216	1.551 ± 0.072	2.559 ± 0.229	1.916 ± 0.290	0.893 ± 0.225	0.929 ± 0.062
Wild type	0.945 ± 0.103	1.158 ± 0.083	0.920 ± 0.079	1.785 ± 0.192	0.802 ± 0.120	1.019 ± 0.030

Table S7. Number of plants exhibiting spontaneous systemic silencing under blue and red light conditions over the total number of plants examined (6.4 line, ≤5 leaf stage).

**Light conditions**	**Number of plants exhibiting systemic silencing / total number of plants examined (%)**
Blue Light	6/27 (22%)
Red Light	4/32 (13%)

Table S8. Number of plants exhibiting spontaneous short-range silencing (SSRS) under blue and red light conditions over the total number of plants examined (6.4 line, ≤5 leaf stage).

Light conditions	Number of plants exhibiting SSRS / total number of plants examined (%)
Blue Light	3/27 (11%)
Red Light	0/32 (0%)

Table S9. List of primer sequences used in quantitative real-time PCR assays.

Primer ID	Primer sequence 5´- 3´
DCL1_FOR	TGTGGGTGATGCAGTATT
DCL1_REV	TGAAACCTGGTTTTGATAGT
DCL2_FOR	CGGGATCC-CCGGGATTTATTCGTAAT
DCL2_REV	CCCTCGAG-AATGACAAAGCCGCTACT
DCL3_FORa	ACTTGTTGAATGCGGTGAAG
DCL3_REVa	CCCCTGTCGTTCTAGCTCAT
DCL3_FORb	GGGGAGGAATTGAATCTTCTG
DCL3_REVb	TCCAGTATGAATGGCCACAA
DCL4_FORa	CGTCCGTGCCCAGAAATCT
DCL4_REVa	AATGCAATTGCCGCTTTGA
DCL4_FORb	GCACTTACTACAGAGAAATGCAATG
DCL4_REVb	ACAATGTTTGAGCGCCTTCT
AGO1_FOR	GCTCTAGAAGATCTGTACAAGACTTGGC
AGO1_REV	CGAATTCTTATTGGCAAACAACCTAGT
RDR6_FOR	CTTTGGATGAGAAGTGCCTA
RDR6_REV	TTTGGGACAAGCTCAAGTC
UBI3_FOR*	GCCGACTACAACATCCAGAAGG
UBI3_REV*	TGCAACACAGCGAGCTTAACC
EF1_FOR*	GATTGGTGGTATTGGAACTGTC
EF1_REV*	AGCTTCGTGGTGCATCTC

*These primers sequences were used in Rotenberg *et al.* (2006).
